# Whole exome sequencing identifies recessive germline mutations in *FAM160A1* in familial NK/T cell lymphoma

**DOI:** 10.1038/s41408-018-0149-5

**Published:** 2018-11-12

**Authors:** Jason Yongsheng Chan, Alvin Yu Jin Ng, Chee Leong Cheng, Maarja-Liisa Nairismägi, Byrappa Venkatesh, Daryl Ming Zhe Cheah, Shao-Tzu Li, Sock Hoai Chan, Joanne Ngeow, Yurike Laurensia, Jing Quan Lim, Jane Wan Lu Pang, Sanjanaa Nagarajan, Tammy Song, Burton Chia, Jing Tan, Dachuan Huang, Yeow Tee Goh, Eileen Poon, Nagavalli Somasundaram, Miriam Tao, Richard Hong Hui Quek, Mohamad Farid, Chiea Chuen Khor, Jin-Xin Bei, Soo Yong Tan, Soon Thye Lim, Choon Kiat Ong, Tiffany Tang

**Affiliations:** 1 Division of Medical Oncology, National Cancer Centre Singapore, Singapore, Singapore; 2 Cancer Science Institute of Singapore, National University of Singapore, Singapore, Singapore; 3 SingHealth Duke-NUS Blood Cancer Centre, Singapore, Singapore; 4 Comparative and Medical Genomics Laboratory, Institute of Molecular and Cell Biology, A*STAR, Singapore, Singapore; 5 Department of Anatomical Pathology, Singapore General Hospital, Singapore, Singapore; 6 Lymphoma Genomic Translational Research Laboratory, Division of Cellular and Molecular Research, National Cancer Centre Singapore, Singapore, Singapore; 7 Department of Paediatrics, Yong Loo Lin School of Medicine, National University of Singapore, Singapore, Singapore; 8 Cancer Genetics Service, Division of Medical Oncology, National Cancer Centre Singapore, Singapore, Singapore; 9 Lee Kong Chian School of Medicine, Nanyang Technological University, Nanyang Ave, Singapore; 10 Duke-NUS Medical School, Singapore, Singapore; 11 Department of Hematology, Singapore General Hospital, Singapore, Singapore; 12 Genome Institute of Singapore, A*STAR, Singapore, Singapore; 13 Singapore Eye Research Institute, Singapore, Singapore; 14 State Key Laboratory of Oncology in South China, Collaborative Innovation Center for Cancer Medicine, Sun Yat-Sen University Cancer Center, Guangzhou, P. R. China; 15 Center for Precision Medicine, Sun Yat-Sen University, Guangzhou, P. R. China; 16 Department of Pathology, National University Hospital, National University Health System, Singapore, Singapore; 17 Advanced Molecular Pathology Laboratory, Institute of Molecular and Cell Biology, A*STAR, Singapore, Singapore

## Introduction

Natural-killer/T-cell lymphoma (NKTL) is a rare subset of non-Hodgkin lymphoma that demonstrates a unique geographic distribution, with higher prevalence in Asia compared to the West^1^. While cure remains achievable in early-stage disease, the prognosis for advanced-stage NKTL is dismal^2^. Recently, some progress has been made in uncovering the molecular pathogenesis of NKTL. In a genome-wide association study, strong correlations between *HLA-DPB1* single-nucleotide polymorphisms and NKTL susceptibility were discovered, implicating altered antigen processing and presentation to CD4-positive T-lymphocytes in this Epstein-Barr virus (EBV)-associated malignancy^3^. Next generation sequencing also revealed recurrent somatic mutations such as *TP53*, *JAK3*, *STAT3* and *DDX3X* in NKTL^4,5^. In this paper, we report a pair of male siblings from a non-consanguineous Chinese family who were diagnosed with NKTL, and provide initial evidence for novel recessive germline mutations in *FAM160A1* identified through next-generation sequencing.

The index patient was 35 years old, when he presented with nasal blockage in March 2013. 18-FDG-PET/CT imaging revealed an 18-FDG-avid nasal mass infiltrating into the palate, as well as enlarged cervical lymph nodes. Biopsy of the mass showed abnormal lymphoid cells positive for CD56 by immunohistochemistry (IHC) as well as EBV-encoded RNA (EBER) by in-situ hybridization. He was diagnosed with stage IIA extranodal NKTL, nasal type, and treated with 4 cycles of bortezomib-GIFOX (gemcitabine, ifosfamide and oxaliplatin) as part of a clinical trial followed by radiotherapy to the nasal region. He had primary-refractory disease and was further treated with 4 cycles of SMILE (dexamethasone, methotrexate, ifosfamide, l-asparaginase, and etoposide) followed by high-dose chemotherapy and autologous stem cell transplantation. He progressed and received ruxolitinib off-label, followed by RAD001 (mTOR inhibitor) and LBH589B (histone deacetylase inhibitor) as part of another clinical trial. He then had radiotherapy to an ulcerating penile lesion before he died of progressive disease 27 months after diagnosis (Supplementary Table 1).

**Fig. 1:**
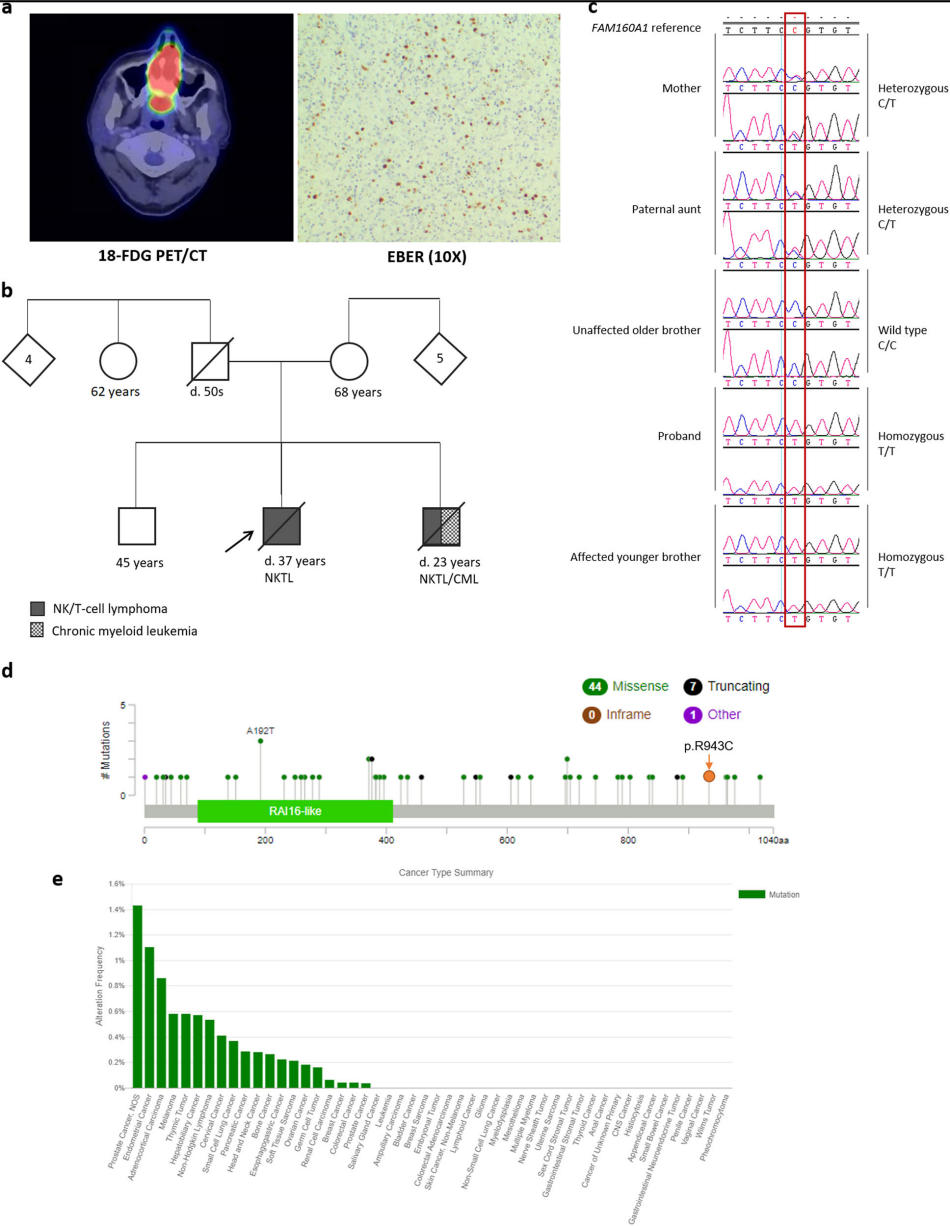
Clinical characterization of brothers with familial NKTL. **a** 18-FDG-PET/CT image depicting a large nasal mass infiltrating into the palate, of which biopsy showed abnormal lymphoid cells positive for Epstein-Barr virus encoded RNA (EBER) by in situ hybridization. **b**, **c** Inheritance modelling identified homozygous germline mutations of *FAM160A1* c.2827 C > T in both affected brothers, heterozygous carriage in their mother and paternal aunt, and homozygous wildtype in their healthy older brother. **d**, **e** The non-synonymous substitution at *FAM160A1* c.2827 C > T results in an amino-acid alteration from arginine to cysteine (p.R943C). Patterns and frequencies of known mutations in other cancer types are shown

His younger brother was 18 years old, when diagnosed with NKTL affecting the nasal floor in 1998 following a bout of epistaxis. He received 6 cycles of CHOP (cyclophosphamide, doxorubicin, vincristine, and prednisone) with high-dose methotrexate and went into complete remission. Three years later he was diagnosed with chronic myeloid leukemia, for which he received hydroxyurea. In 2004, he had a relapse of NKTL and received ESHAP (etoposide, prednisolone, cytarabine and cisplatin), total body irradiation and allogeneic bone marrow transplant. He died of transplant-related complications 6 years from diagnosis. These siblings have an older brother unaffected by any hematological malignancy. Their father died of a non-malignant condition in his fifties and their mother remains well at the age of 68 years. Among their first-degree relatives, none were known to be inflicted with hematologic malignancies (Fig. 1).

Despite recent improvements in the understanding of familial lymphoma, the specific predisposition genetic factors remain largely unknown^6^. To the best of our knowledge, familial cases of NKTL are extremely rare occurrences. Previously, a familial occurrence of nasal NKTL in a father and son pair with known heavy pesticide exposure had been reported in 2001, but no specific genetic element was evident^7^. In another study, biallelic truncating *MSH2* mutations had been described in familial T-cell non-Hodgkin lymphoma affecting three siblings^8^. In order to identify potential pathogenic germline mutations that contribute to NKTL in the affected siblings, we performed whole exome sequencing on DNA samples obtained from both of them as well as their unaffected family members (older brother and mother). As both parents of the affected siblings were unaffected by NKTL, only autosomal recessive and x-linked recessive models were explored. Details regarding sample curation, next generation sequencing, bioinformatics as well as pathological analyzes are described in the Supplementary section. Using the x-linked recessive model, a single nucleotide variant located in the 3’-untranslated region (UTR) of *HNRNPH2* (X-100668489-T-G, rs41307260) was initially detected. This particular variant however, had low coverage (14X) and subsequently was not observed on Sanger sequencing. Out of 39 candidate variants identified in the autosomal recessive model, one was in the 5’-UTR (*RDH13*), three in the 3’-UTR (*GP6, NLRP2, LILRB3*), and one in the coding exon (*FAM160A1* c.2827C > T). These five variants were verified by Sanger sequencing (Supplementary Figure 1). Given the extreme rarity of familial NKTL, it is unlikely that any pathogenic variant would present as a common polymorphism in the population (minor allele frequencies: *RDH13*, 0.2101; *GP6*, 0.7535; *NLRP2*, 0.3729; *LILRB3*, 0.05749), therefore leaving only *FAM160A1* as the remaining candidate (minor allele frequency: 0.00001892). Both affected brothers were *FAM160A1* homozygous mutants, their mother was a heterozygous carrier, and their older unaffected brother was homozygous wild-type. In keeping with this result, direct sequencing of peripheral-blood DNA of their paternal aunt revealed heterozygous *FAM160A1* c.2827C/T (Fig. 1).

The non-synonymous substitution at *FAM160A1* c.2827C > T results in an amino acid alteration from arginine to cysteine (p.R943C), and is predicted to be deleterious based on multiple in-silico algorithms including PROVEAN (prediction score, −4.74), SIFT (prediction score, 0.000), PolyPhen-2 (prediction score, 1.000), MutationAssessor (prediction score, 2.99), MutationTaster, and M-CAP (See Supplementary Tables 2–6 and References).

Somatic variants detected in the proband included 66 non-silent mutations, including those previously described in NKTL such as *DDX3X*, *STAT3*, and *PRDM1*^4^. Specifically, stopgain mutations in *DDX3X* c.G1639T (p.E547X), missense mutations in *STAT3* c.G1981C (p.D661H), and *PRDM1* c.C1877T (p.T626M) were observed. Additionally, within limitations of the analysis, an attempt to identify somatic variants in the affected younger brother revealed 192 potential non-silent mutations (Supplementary Table 7).

**Fig. 2:**
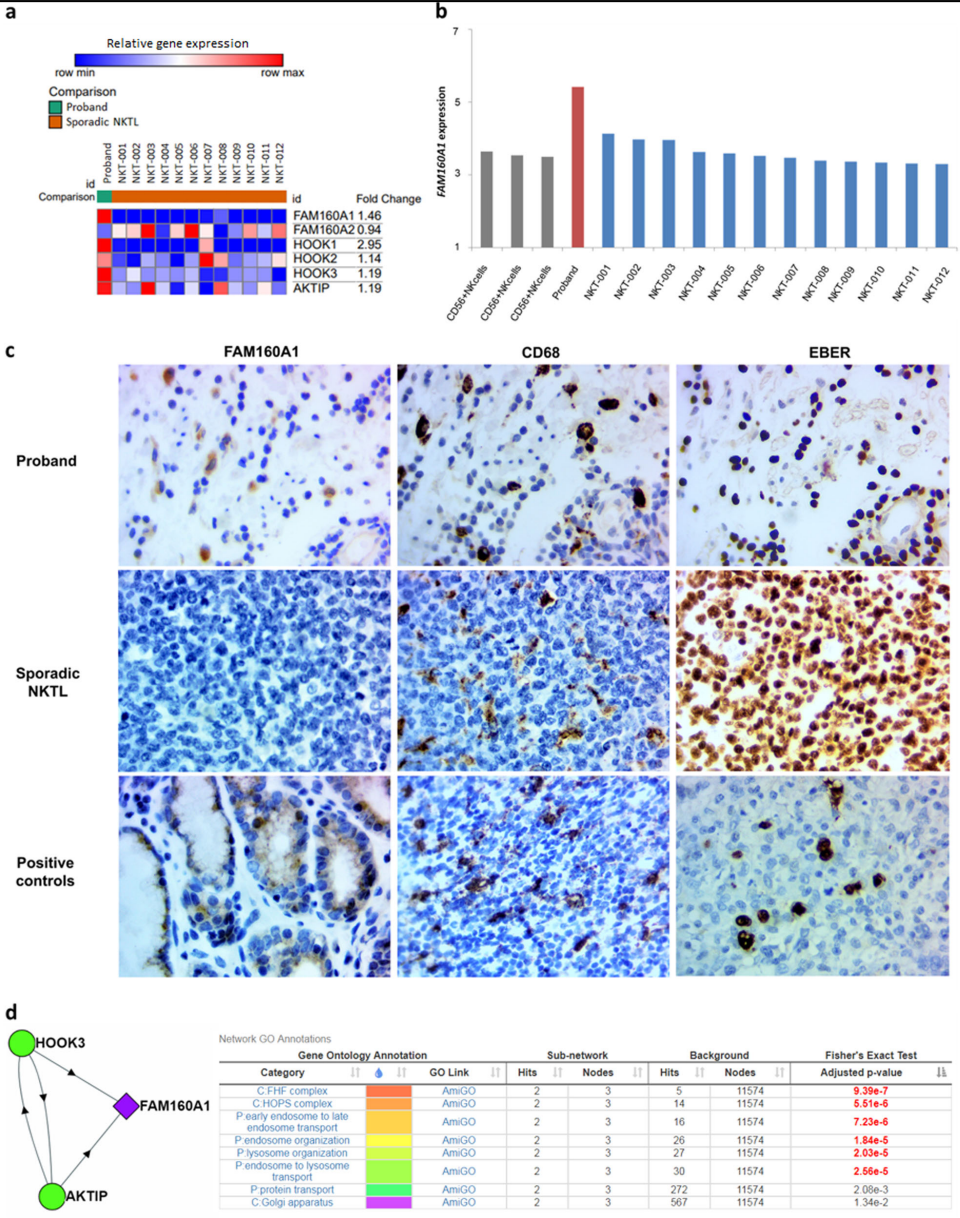
Overexpression of FAM160A1 in the proband’s NKTL tissue compared to sporadic NKTL, as shown on **a** GeneChip Array, **b** real-time PCR, and **c** immunohistochemistry. Strong cytoplasmic staining was observed in tumor-infiltrating histiocytes positive for CD68. Human interactome and gene ontology analyzes suggest significant interaction of FAM160A1 with AKTIP and HOOK3 (both members of the FTS/Hook/FHIP complex). A relative increase in expression of AKTIP and Hook-family genes were also observed

Gene expression profiling revealed that *FAM160A1* was significantly overexpressed in the index patient’s tumor tissue compared to sporadic NKTL (*n* = 12) (Fig. 2). In keeping with this finding, strong cytoplasmic FAM160A1 staining by IHC was observed in scattered tumor-infiltrating cells, which corresponded to CD68-positive histiocytes. Lymphomatous cells, which were EBER-positive, did not stain positive for FAM160A1. This staining pattern was consistently observed in independent samples of metachronous skin metastases over the clavicle and foreskin (Supplementary Figure 2). Among the sporadic NKTLs with available tissue for IHC (*n* = 14), two displayed weak-cytoplasmic staining for FAM160A1, while the rest were negative. Examination of mutation frequencies in sporadic NKTL cases, derived from Singapore (*n* = 33) and Chinese cohorts (*n* = 189) in a previously published dataset^3^, did not reveal any *FAM160A1* c.2827C > T variants. Furthermore, a preliminary analysis of our in-house clinical dataset of sporadic NKTL suggests that higher expression of *FAM160A1* did not lead to differences in survival outcomes (data not shown). Taken together, this indicates that alterations in *FAM160A1* may be unique to familial rather than sporadic NKTL.

FAM160A1 is a member of the UPF0518 family of proteins, each containing a conserved retinoic acid induced 16 (RAI16)-like domain with unknown biological function. Other members of this family include FAM160A2, FAM160B1, and FAM160B2. In particular, FAM160A2 (also known as FHIP), is a component of the FTS/Hook/FHIP complex (FHF complex), which functions to promote vesicle trafficking and/or fusion via the homotypic-vesicular protein sorting complex (the HOPS complex)^9^. In terms of its mutation frequency in cancer tissues, querying 35976 cancer samples across 150 studies revealed only 52 somatic point mutations (44 missense, 7 truncating, and 1 non-start) scattered across the gene^10^. Analysis of the human interactome^11^ and gene ontology^12^ revealed significant interactions of FAM160A1 with AKT-interacting protein (AKTIP, also known as FTS) and Protein Hook homolog 3 (HOOK3) (adjusted *p*-value 9.39e−7)—both components of the FTS/Hook/FHIP (FHF) complex^9^, suggesting that FAM160A1 may be part of this complex as well. A relative increase in expression of genes encoding for members of the FHF complex, including AKTIP (1.19 fold), HOOK3 (1.19 fold), HOOK2 (1.14 fold), HOOK1 (2.95 fold), but not FAM160A2 (also known as FHIP) (0.94 fold), was observed.

Our current findings not only reinforce the existence of familial T and NK-cell lymphomas as per previous reports^7,8^, but, in addition, provide evidence for a novel genetic basis that may explain this rare phenomenon. The importance of delineating a detailed family history, in addition to discriminating usage of next-generation sequencing cannot be further emphasized, in order for our current findings to be confirmed in future cohorts of patients.

In summary, this report implicates a novel recessive germline mutation in *FAM160A1* with familial NKTL.

## Electronic supplementary material

Supplementary Files

Supplementary Table 3

Supplementary Table 7
