# Supplementary material for: Whole exome sequencing identifies recessive germline mutations in FAM160A1 in familial NK/T cell lymphoma
Source: Blood Cancer J. 2018 Nov 12;8(11):111. doi: 10.1038/s41408-018-0149-5 (PMC6232147; doi:10.1038/s41408-018-0149-5)
Supplement: Supplementary file 1 — Supplementary Files [file 41408_2018_149_MOESM1_ESM.docx]

**SUPPLEMENTARY METHODS**

**Patient data and specimens**

All clinical information of patients affected with NKTL, including the affected pair of siblings were retrieved from electronic medical records. Demographic data including sex, age and ethnicity of the affected siblings and their family members were verified against their National Registry Identification Cards. All patients obtained their initial diagnosis and received primary treatment at the Singapore General Hospital and at the National Cancer Centre Singapore. All histological parameters were reviewed by expert hematolymphoid pathologists. Written consent for use of biospecimens and clinical data were obtained in accordance with the Declaration of Helsinki. Tissue collection and consent protocols were performed as part of the Singapore Lymphoma Study and were under approval from the SingHealth Centralized Institution Review Board.

**Genomic DNA extraction**

Genomic DNA from mouse tumor xenografts and peripheral whole blood samples were available from the index patient. For his affected younger brother, DNA was available only from snap-frozen tumor obtained via biopsy of liver metastases. Genomic DNA from their mother, older brother and paternal aunt were obtained from peripheral blood samples. No material was available from their deceased father. Genomic DNA was extracted from tissue and blood specimens using the Blood & Cell Culture DNA Kit (Qiagen, Hilden, Germany). Genomic DNA yield and quality were determined by Quant-i PicoGreen dsDNA Assay Kit (Invitrogen, Carlsbad, CA, USA) and Nanodrop 1000 spectrophotometer (Thermo Scientific, Wilmington, DE, USA), and visually inspected by agarose gel electrophoresis.

**Library construction, whole exome sequencing and bioinformatic analyses**

We performed whole exome sequencing on paired tumor and normal DNA from both the index patient, tumor-only DNA from his affected younger brother, as well as DNA (from whole blood) of their healthy older brother and mother. Two micrograms of genomic DNA was sheared to 200 bp using a Covaris E Series ultrasonic solubilizer (Covaris Inc., Woburn, MA, USA) and after adapter ligation the resultant library was subjected to DNA capture based on the Agilent SureSelect Human All Exon platform (Agilent, Santa Clara, CA, USA). Library construction and DNA capture were carried out according to the manufacturer’s instructions. Captured libraries were sequenced on a HiSeq 2000 platform (Illumina, San Diego, CA, USA). Paired-end reads were aligned to the National Center for Biotechnology Information (NCBI) human reference genome GRCh37 (hg19) using BWA-MEM software.^1^ PCR duplicates removal was done using SAMtools.^2^ The Genome Analysis Tool Kit (GATK)^3^ was used for base quality score recalibration, local realignment around indels, and variant calling.

The VCF files generated from tumor-normal samples of the index patient, older brother and mother were imported and annotated in Ion Reporter v4.2 (<https://ionreporter.thermofisher.com/>). Out of a total of 3.03 million variants detected before quality control, a total of 484,385 candidate variants were considered among the 4 samples. Each variant was annotated with the associated gene, location, quality-score, coverage, predicted functional consequences, protein position and amino acid changes; SIFT^4^, PolyPhen2^5^ and Grantham^6^ prediction scores, phyloP conservation scores^7^ and 5000 genomes Minor Allele Frequencies. Variants were filtered for common SNPs using the NCBI’s “common and no known medical impacts” database (<ftp://ftp.ncbi.nlm.nih.gov/pub/clinvar/vcf_GRCh37/>) and the minor allele frequency of < 1% Exome Aggregation Consortium (ExAC) database (<ftp://ftp.broadinstitute.org/pub/ExAC_release/release0.2/>). The variants selected were further filtered by >10X coverage. Variants that were predicted to be synonymous or not having a location on either a coding exon, UTR, splice site junction or flanking intron were filtered out. Next, we applied filters to retain the index patient’s variants that follow the autosomal recessive and x-linked inheritance models. In the autosomal recessive model, both affected siblings would be homozygous for the mutation, both their parents would be assumed heterozygous carriers, and their older brother either also a heterozygous carrier or homozygous wild-type. In the x-linked recessive model, both affected siblings would have inherited the implicated allele from their healthy mother, who would be a heterozygous carrier. Their father and older brother would be assumed wild-type. A final set of 39 candidate variants for the autosomal recessive model and 9 candidate variants for the x-linked model remained. All candidate variants were visually inspected in the Integrative Genomics Viewer.^8^

**Somatic variant identification**

To detect somatic variants from the proband’s tumor, reads derived from mouse tumor xenografts were first processed *in silico* to separate host reads from contaminant mouse reads using Xenome.^9^ Reads were aligned to mouse reference genome mm10 and human reference genome hg19lite, with 6.70% and 92.07% of read counts corresponding to the mouse and human genome, respectively (Supplementary Table 8). More than 95% of targeted regions were covered by ≥ 20X read-depth. Somatic variants were called using Strelka.^10^ For the affected younger brother, as no paired normal blood or tissue were available, single-sample callings by Freebayes^11^ were performed, and filtered using blood samples from his unaffected brother and mother. Only single nucleotide variants that passed additional quality filters (minor allele frequency < 0.5%, variant allele frequency ≥ 10%, and total depth ≥ 20X) were retained.

**Sanger sequencing validation**

To validate candidate germline variants by Sanger sequencing, PCR amplicons encompassing the mutation sites were sequenced using the ABI PRISM BigDye Terminator Cycle Sequencing Ready Reaction kit (Applied Biosystems, Foster City, CA, USA) on an ABI 3730 xl DNA Analyzer (Applied Biosystems). PCR primers for amplification and sequencing (Supplementary Table 9) were designed by targeting regions immediately flanking the predicted variant using Primer3 software.^12^

**cDNA synthesis, real-time PCR and gene expression profiling**

Total RNA was extracted using TRIzol (Invitrogen, Carlsbad, CA, USA) and purified with RNeasy Mini Kit (Qiagen, Hilden, Germany) according to manufacturer’s instructions. The integrity of RNA was determined by electrophoresis using 2100 Bioanalyzer (Agilent Technologies, Palo Alto, CA, USA). Total RNA (500 ng) was reverse transcribed with iScript cDNA Synthesis Kit (Bio-Rad, Hercules, CA, USA). Quantification was performed using SsoFast EvaGreen Supermix (Bio-Rad) and CFX96 Real-Time PCR System (Bio-Rad). Primer sequences were: forward, GCTGCTTATCGGGATCATTA; reverse, CACACGGAAGAGCAGGTACT. Relative quantification of mRNA levels was calculated using the ΔCt-method. Real-time PCR for *FAM160A1* was performed on a snap frozen NKTL tumor sample (skin metastasis) from the proband, sporadic NKTLs from 12 patients (Supplementary Table 10), as well as isolated CD56+ NK cells from 3 healthy volunteers. The NKTL samples were also analysed on the GeneChip Human Genome U133 Plus 2.0 Array (Affymetrix, Santa Clara, CA, USA) as previously described.^13^ Analysis of the human interactome and gene ontology was performed on Bioplex 2.0^14^ and the expression profile of selected genes were represented as heat maps generated using Morpheus (Morpheus, https://software.broadinstitute.org/morpheus).

**Tissue microarray construction, immunohistochemistry and *in situ* hybridization**

Tissue microarray (TMA) containing representative 1 mm cores from 14 NKTL samples were punched out from each tissue block and deposited into a recipient TMA block using a tissue arrayer. Multiple sections (4 μm) were cut from the TMA blocks and mounted onto positively-charged Bond Plus Slides (Leica Biosystems, Inc., Richmond, IL, USA) glass slides, and dried on a heating bench for at least 20 minutes. The sections were run on a Bond III automated stainer (Leica Biosystems). After deparaffinization and rehydration, tissue samples were subjected to high temperature-induced epitope retrieval by briefly steaming them in Bond Epitope Retrieval Solution (ER1 for FAM160A1 and ER2 for CD68) at 98°C for 20 minutes. The detection system used was the Bond Polymer Refine Detection Kit DS9800 (Leica Biosystems). Peroxidase blocking was carried out for 10 min. Rabbit polyclonal IgG1 antibody (Sigma-Alrich, St. Louis, MO, USA) was used to detect FAM160A1 (1:200 dilution) and mouse anti-human monoclonal antibody (Dako) was used to detect CD68 (Clone PG-M1, 1:100 dilution). Slides were incubated with the primary antibody for 30 minutes at room temperature. This was followed by incubation with polymer reagent, addition of substrate chromogen, diaminoazobenzidine (DAB) for 5 minute, and counterstaining with hematoxylin. Detection of EBV-encoded RNA (EBER) for latent EBV infection was performed using the Bond™ Ready-to-Use ISH EBER Probe (probe PB0589, Leica Microsystems) according to manufacturer’s instructions. Appropriate controls were run with each batch of slides. Positive controls for FAM160A1 consisted of human stomach demonstrating distinct cytoplasmic positivity in glandular cells, while negative controls consisted of samples of diffuse large B cell lymphoma. A moderate to strong staining pattern was regarded as positive, while absent to weak staining was taken as negative. Human appendix with infiltrating histiocytes were used as positive controls for CD68 PG-M1.

**SUPPLEMENTARY REFERENCES**

1. Li H. Aligning sequence reads, clone sequences and assembly contigs with BWA-MEM. arXiv:1303.3997v2.
2. Li H, Handsaker B, Wysoker A, Fennell T, Ruan J, Homer N *et al*. The Sequence Alignment/Map format and SAMtools. *Bioinformatics* 2009; **25**: 2078–2079.
3. McKenna A, Hanna M, Banks E, Sivachenko A, Cibulskis K, Kernytsky A *et al*. The Genome Analysis Toolkit: a MapReduce framework for analysing next-generation DNA sequencing data. *Genome Res* 2010; **20**: 1297–1303.
4. Kumar P, Henikoff S, Ng PC. Predicting the effects of coding non-synonymous variants on protein function using the SIFT algorithm. *Nat Protoc* 2009; **4**: 1073-1081.
5. Adzhubei IA, Schmidt S, Peshkin L, Ramensky VE, Gerasimova A, Bork P *et al.* A method and server for predicting damaging missense mutations. *Nat Methods* 2010; **7**: 248-249.
6. Grantham R. Amino acid difference formula to help explain protein evolution. *Science* 1974; **185**: 862-864.
7. Pollard KS, Hubisz MJ, Rosenbloom KR, Siepel A. Detection of nonneutral substitution rates on mammalian phylogenies. *Genome Res* 2010; **20**: 110-121.
8. Robinson JT, Thorvaldsdóttir H, Wenger AM, Zehir A, Mesirov JP. Variant Review with the Integrative Genomics Viewer. *Cancer Res* 2017; **77**: e31-e34.
9. Conway T, Wazny J, Bromage A, Tymms M, Sooraj D, Williams ED *et al.* Xenome--a tool for classifying reads from xenograft samples. *Bioinformatics* 2012; **28**: i172-178.
10. Saunders CT, Wong WS, Swamy S, Becq J, Murray LJ, Cheetham RK. Strelka: accurate somatic small-variant calling from sequenced tumor-normal sample pairs. *Bioinformatics* 2012; **28**: 1811-1817.
11. Garrison E, Marth G. Haplotype-based variant detection from short-read sequencing. arXiv:1207.3907v2.
12. Untergasser A, Cutcutache I, Koressaar T, Ye J, Faircloth BC, Remm M *et al.* Primer3--new capabilities and interfaces. *Nucleic Acids Res* 2012; **40**: e115.
13. Nairismägi ML, Tan J, Lim JQ, Nagarajan S, Ng CC, Rajasegaran V *et al.* JAK-STAT and G-protein-coupled receptor signaling pathways are frequently altered in epitheliotropic intestinal T-cell lymphoma. *Leukemia* 2016; **30**: 1311-1319.
14. Huttlin EL, Ting L, Bruckner RJ, Gebreab F, Gygi MP, Szpyt J *et al.* The BioPlex Network: A Systematic Exploration of the Human Interactome. *Cell* 2015; **162**: 425-440.
15. Sherry ST, Ward MH, Kholodov M, Baker J, Phan L, Smigielski EM *et al.* dbSNP: the NCBI database of genetic variation. *Nucleic Acids Res* 2001; **29**: 308–311.
16. Lek M, Karczewski KJ, Minikel EV, Samocha KE, Banks E, Fennell T *et al.* Analysis of protein-coding genetic variation in 60,706 humans. *Nature* 2016; **536**: 285-291.
17. Choi Y, Sims GE, Murphy S, Miller JR, Chan AP. Predicting the functional effect of amino acid substitutions and indels. *PLoS One* 2012; **7**: e46688.
18. Kumar P, Henikoff S, Ng PC. Predicting the effects of coding non-synonymous variants on protein function using the SIFT algorithm. *Nat Protoc* 2009; **4**: 1073-1081.
19. Adzhubei IA, Schmidt S, Peshkin L, Ramensky VE, Gerasimova A, Bork P *et al.* A method and server for predicting damaging missense mutations. *Nat Methods* 2010; **7**: 248-249.
20. Reva B, Antipin Y, Sander C. Predicting the functional impact of protein mutations: application to cancer genomics. *Nucleic Acids Res* 2011; **39**: e118.
21. Schwarz JM, Cooper DN, Schuelke M, Seelow D. MutationTaster2: mutation prediction for the deep-sequencing age. *Nat Methods* 2014; **11**: 361-362.
22. Jagadeesh KA, Wenger AM, Berger MJ, Guturu H, Stenson PD, Cooper DN *et al.* M-CAP eliminates a majority of variants of uncertain significance in clinical exomes at high sensitivity. *Nat Genet* 2016; **48**: 1581-1586.

**
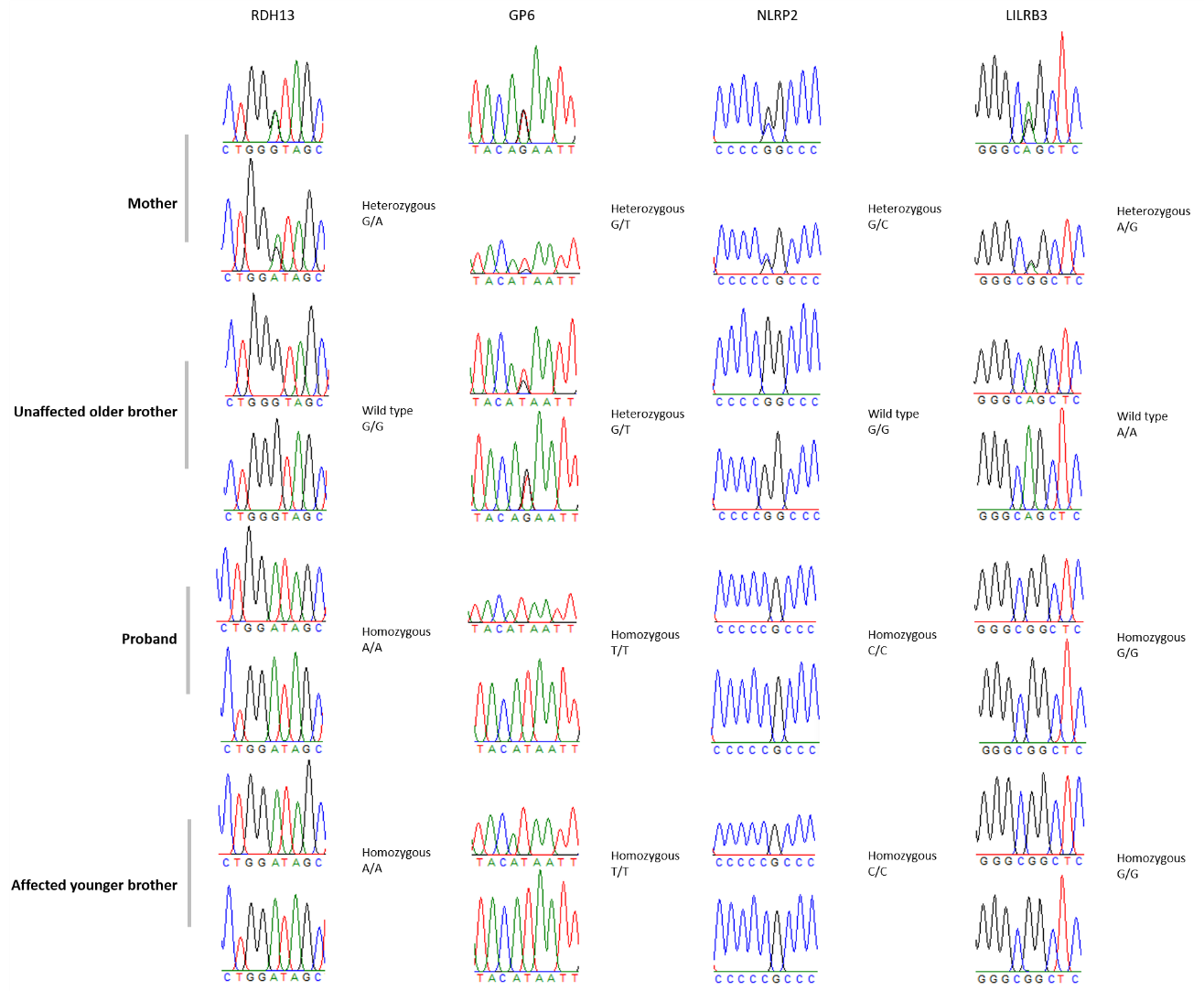
**

**Supplementary Figure 1. Sanger sequencing of candidate variants** in the untranslated regions (UTR) identified in the autosomal recessive model – including 1 in the 5’ UTR (*RDH13*) and 3 in the 3’-UTR (*GP6, NLRP2, LILRB3*).

**
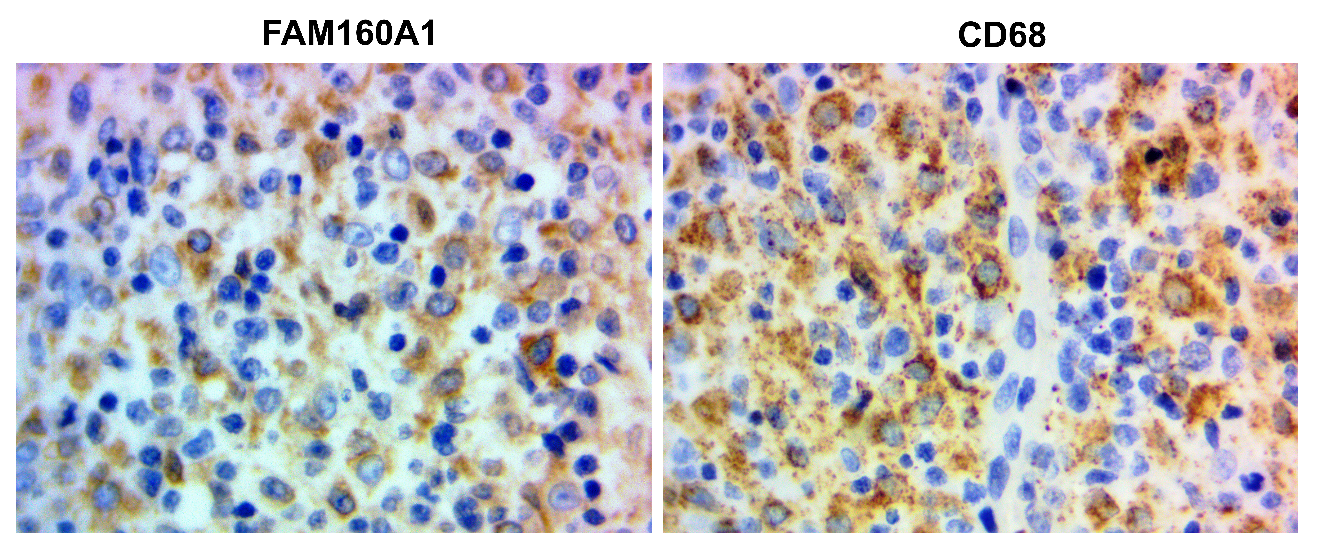
**

**Supplementary Figure 2. FAM160A1 immunochemistry in the proband’s NKTL.** Strong cytoplasmic FAM160A1 staining was observed in scattered tumor-infiltrating cells within a foreskin metastasis of the proband, which corresponded to CD68-positive histiocytes.

**Supplementary Table 1. Clinicopathologic characteristics of brothers affected with NK/T cell lymphoma**

| Characteristics | Index patient | Affected younger brother |
| --- | --- | --- |
| Age at diagnosis (years) | 35 | 18 |
| Age at death (years) | 37 | 23 |
| Site of disease | Nasal floor, cervical lymph nodes | Nasal floor |
| Stage (Ann Arbor) | IIE | IE |
| Other significant diagnoses | Nil | CML at age 21 treated with hydroxyurea |
| Treatment | Bortezomib-GIFOX trial  Nasal radiotherapy  SMILE  HDC - Autologous transplant  Ruxolitinib  RAD001/LBH589B trial  Penile radiotherapy | CHOP-M  ESHAP  TBI - Allogeneic transplant |

Abbreviations: GIFOX, gemcitabine, ifosfamide oxaliplatin; SMILE, dexamethasone, methotrexate, ifosfamide, l-asparaginase, etoposide; HDC, high-dose chemotherapy; HDAC, histone deacetylase; CHOP-M, cyclophosphamide, doxorubicin, vincristine, prednisone, methotrexate; ESHAP, etoposide, prednisolone, cytarabine, cisplatin; TBI, total body irradiation

**Supplementary Table 2. Summary of samples sequenced and mapping statistics**

| Sample source | Coverage  (average) | Sure Select Capturing Kit | Variants selected for analysis |
| --- | --- | --- | --- |
| Index patient  Tumor*  Blood | 138.3  106.5 | V4 + UTR  V4 + UTR | 94,590 |
| Affected younger brother  Tumor** | 187.9 | V5 | 203,771 |
| Unaffected older brother  Blood | 73.2 | V5 | 94,657 |
| Mother  Blood | 72.4 | V5 | 91,367 |
| Paternal aunt  Blood | NA | NA | Sanger sequencing only |

* From mouse patient-derived xenograft tissue used for somatic mutation analysis only

** DNA isolated from snap frozen tumor tissue used for germline analysis as no normal tissue or blood available

**Supplementary Table 3. Set of 39 candidate variants for the autosomal recessive model and 9 candidate variants for the x-linked recessive model.**

(See excel file)

**Supplementary Table 4. Candidate variants identified in autosomal recessive model**

| **Candidate gene** | **Coverage (X)** | **Gene coordinates** | **dbSNP rsID** | **Minor allele frequency*** |
| --- | --- | --- | --- | --- |
| **5’-UTR**  *RDH13* | 57 | 19-55574468-G-A | rs1654458 | 0.2101 |
| **3’-UTR**  *GP6*  *NLRP2*  *LILRB3* | 80  33  97 | 19-55525388-G-T  19-55512347-G-C  19-54720896-A-G | rs10417981  rs1043680  rs3745410 | 0.7535  0.3729 0.05749 |
| **Exonic**  *FAM160A1* | 142 | 4-152578008-C-T | rs551441542 | 0.0001892 |

*Based on Gnomad database

**Supplementary Table 5. Frequency of *FAM160A1* c.2827C>T variants in existing databases**

| **Database** | **Allele count** | **Allele number** | **Minor allele frequency** | **Reference** |
| --- | --- | --- | --- | --- |
| **dbSNP**  East Asian  European  African  American  South Asian | 1  0  0  0  0 | 1008  1006  1322  694  978 | 0.0009921  0.000  0.000  0.000  0.000 | [15] |
| **ExAC**  East Asian  European (non-Finnish)  European (Finnish)  African  Latino  South Asian  Other | 1  1  0  0  0  0  0 | 622  8432  34  2148  410  78480  1880 | 0.001608  0.0001186  0.000  0.000  0.000  0.000  0.000 | [16] |
| **Gnomad**  East Asian  European (non-Finnish)  European (Finnish)  African  Latino  South Asian  Ashkenazi Jewish  Other | 28  2  0  1  3  0  0  0 | 11860  71982  18544  16336  24790  22816  8536  4800 | 0.0002361  0.00002778  0.000  0.00006121  0.0001210  0.000  0.000  0.000 | [16] |

*No homozygote variants were identified in any database

**Supplementary Table 6. Predicted functional impact of *FAM160A1* c.2827C>T**

| **Algorithm** | **Prediction score** | **Predicted functional impact** | **Reference** |
| --- | --- | --- | --- |
| PROVEAN | -4.74 | Deleterious | [17] |
| SIFT | 0.000 | Damaging | [18] |
| PolyPhen-2 | 1.000 | Probably damaging | [19] |
| MutationAssessor | 2.99 | Medium | [20] |
| MutationTaster | NA | Disease causing | [21] |
| M-CAP | 0.049 | Possibly Pathogenic | [22] |

**Supplementary Table 7. Coding somatic mutations in both NKTL samples from affected siblings**

(See excel file)

**Supplementary Table 8. Separation of host-graft reads**

| **Read counts** | **Percentage** | **Class** |
| --- | --- | --- |
| 75467242 | 92.07 | Human |
| 5490462 | 6.70 | Mouse |
| 29216 | 0.04 | Both |
| 39393 | 0.05 | Neither |
| 939680 | 1.15 | Ambiguous |

**Supplementary Table 9. Primer sequences for Sanger sequencing**

| Target genes | Primer sequences |
| --- | --- |
| FAM160A1 | Forward: GGAGTGGAATTTCATGGTGAGG  Reverse: TTCTAGGATCCAGAAGCCAGTG |
| RDH13 | Forward: CGGGGCTAGAATGTACTCACTT  Reverse: GATGCTCATAGGTCCCGTCC |
| GP6 | Forward: GGTCAAGAAATGCCTAAACGCT  Reverse: CATCCCTATACTCCCAGCAACT |
| NLRP2 | Forward: ACTGAGAAACATCATCCCTGGG  Reverse: TCAATGCAATTAAACACTGTCACT |
| LILRB3 | Forward: CCGGCCTTTACGTCTGTTTT  Reverse: CCACCACGTTCCTTACCTCT |
| HNRNPH2 | Forward: GCTTAGGTAGAGAAGGAGCACT  Reverse: AGTGTTCAAAATTACTGAGCTTGA |

**Supplementary Table 10. Clinicopathologic characteristics of sporadic NKTL used for gene expression profiling (GEP) and immunohistochemistry**

| Patient ID | Age at diagnosis (years) | Stage at diagnosis  (Ann Arbor) | GEP  performed | FAM160A1  Immunohistochemistry |
| --- | --- | --- | --- | --- |
| NKT-001 | 50.3 | IE | Yes | Negative (absent) |
| NKT-002 | 51.1 | IIIE | Yes | Negative (absent) |
| NKT-003 | 73 | I | Yes | Not done |
| NKT-004 | 37.0 | IV | Yes | Not done |
| NKT-005 | 58.3 | I | Yes | Not done |
| NKT-006 | 50.1 | IV | Yes | Not done |
| NKT-007 | 34 | IE | Yes | Not done |
| NKT-008 | 41 | IV | Yes | Not done |
| NKT-009 | 25 | IIE | Yes | Not done |
| NKT-010 | 56 | IV | Yes | Not done |
| NKT-011 | 33 | IIE | Yes | Not done |
| NKT-012 | 36 | IIE | Yes | Not done |
| NKT-013 | 77.9 | IV | No | Negative (weak) |
| NKT-014 | 40.0 | IIE | No | Negative (weak) |
| NKT-015 | 81 | IE | No | Negative (absent) |
| NKT-016 | 25.7 | IIE | No | Negative (absent) |
| NKT-017 | 56.8 | IV | No | Negative (absent) |
| NKT-018 | 49.4 | IV | No | Negative (absent) |
| NKT-019 | 64.9 | IV | No | Negative (absent) |
| NKT-020 | 75.8 | IE | No | Negative (absent) |
| NKT-021 | 61.2 | IV | No | Negative (absent) |
| NKT-022 | 53.2 | IE | No | Negative (absent) |
| NKT-023 | 24.8 | IIE | No | Negative (absent) |
| NKT-024 | 71.9 | IE | No | Negative (absent) |
